# Comprehensive analysis and experimental verification of the role of mitochondrial dynamics-related genes in liver hepatocellular carcinoma

**DOI:** 10.1016/j.gendis.2025.101872

**Published:** 2025-09-26

**Authors:** Yuchen Zhang, Jing Yuan, Ning Zhang, Yang Yang, Chang Liu, Xingyu Jiang, Yue Wu, Xingxing Ma, Yinyin Xie

**Affiliations:** a College of Life Sciences, Anhui Medical University, Hefei, Anhui 230032, China; b Second School of Clinical Medicine, Anhui Medical University, Hefei, Anhui 230032, China; c First School of Clinical Medicine, Anhui Medical University, Hefei, Anhui 230032, China; d Department of Obstetrics and Gynecology, First Affiliated Hospital of Anhui Medical University, Hefei, Anhui 230022, China

Liver hepatocellular carcinoma (LIHC) represents the predominant subtype of liver cancer, which is characterized by high rates of occurrence and fatality, and imposes a significant burden on health systems worldwide.^1^ Mitochondria have garnered significant attention within the context of cancer research as key organelles. Mitochondrial dynamics constitute a part of the mitochondrial quality control system, encompassing mitochondrial fission, fusion, mitophagy, and transport, which are critical for maintaining mitochondrial function and cellular homeostasis.^2^^,^^3^ In cancer, mitochondrial dynamics play a significant role in tumor development, progression, and response to therapy. The balance between fission and fusion is tightly regulated, and dysregulation of these processes is often observed in cancer cells, contributing to their altered metabolism, resistance to apoptosis, and increased invasiveness.^4^ There has been a growing emphasis on mitochondrial dynamics-related genes (MDRGs) in oncology.^5^ However, comprehensive studies exploring the roles of MDRGs in LIHC are relatively rare.

This research investigated the involvement of MDRGs in LIHC. We identified two clusters related to mitochondrial dynamics in LIHC. The high-MDRG cluster was associated with a worse prognosis, a higher level of immune infiltration, and greater tumor proliferation and migration. The LIHC risk signature based on MDRGs exhibited outstanding predictive ability. An online platform was further developed for better assistance in clinical decision-making (mitusml.com:9545). In addition, further single-cell and bioinformatics analyses identified a key gene *CHCHD3* (short for coiled-coil-helix-coiled-coil-helix domain containing 3) and reported its vital functions. *In vitro* experiments revealed that its high expression was closely associated with the increased proliferation and migration of tumor cells. In short, these findings suggest the promising potential of MDRGs in the management and treatment of LIHC.

To explore the role of MDRGs in LIHC, tumor and control samples were derived from The Cancer Genome Atlas (TCGA), Gene Expression Omnibus (GEO), and Genotype-Tissue Expression (GTEx) databases. Differential analysis revealed that 6464 genes were up-regulated and that 4771 genes were down-regulated in LIHC (Fig. 1A). After intersecting with MDRGs, a total of 20 differentially expressed MDRGs were obtained. All the expression levels of these genes were notably increased in LIHC (Fig. 1B). Further assessment revealed that 10 MDRGs were significantly associated with prognosis, with a hazard ratio greater than 1 (Fig. 1C). The Kaplan–Meier curves indicated that individuals exhibiting high expression of MDRGs experienced inferior survival outcomes (Fig. S1). These findings suggest that the increased expression of MDRGs might be closely related to the onset of LIHC. Then, we identified two mitochondrial dynamics-related molecular subtypes of LIHC (Fig. S2A). The cumulative distribution function (CDF) plot, along with the area under the curve, further confirmed superior clustering efficacy at k = 2 (Fig. S2B and S2C). Moreover, when k = 2, each cluster exhibited ideal intra-group consistency. However, at k = 3–6, clusters with lower intra-group consistency emerged, complicating the identification of inter-cluster differences (Fig. S2D). Principal component analysis further demonstrated good discriminability between the clusters (Fig. S2E). The expression levels of 10 MDRGs were all consistently higher in C1 compared with those in C2; therefore, we designated C1 as the high-MDRG cluster and C2 as the low-MDRG cluster (Fig. 1D). The Kaplan–Meier plot revealed a worse prognosis for patients allocated to the high-MDRG cluster (Fig. 1E). We analyzed the patients' clinical information and detected significant differences in stage and grade between the two clusters, with patients in the high-MDRG cluster exhibiting more advanced tumor progression (Table S1). These findings indicated that elevated levels of MDRGs could serve as a significant risk indicator for the exacerbation of tumor and poor outcomes in patients with LIHC. The immunological analysis revealed that in LIHC patients, the abundance of dendritic cells was relatively high, and the relationships between different immune cells were closely intertwined (Fig. S3A and S3B). The high-MDRG cluster had a relatively high level of immune infiltration and immune response, which revealed a close association between MDRGs and the immune microenvironment in LIHC (Fig. 1F; Fig. S3C). Mutation analysis of all the LIHC patients revealed that the mutation from base C to base T was the major form of base mutation (Fig. S4A). The mutation profile disclosed unique gene mutation patterns of the two subtypes, with the high-MDRG cluster exhibiting the highest frequency of *TP53* mutations and the low-MDRG cluster characterized by a greater prevalence of *CTNNB1* mutations (Fig. S4B–S4D). The *TP53* mutation type results in increased tumor cell proliferation, whereas the *CTNNB1* mutation type generally results in well-differentiated tumor cells. Gene Set Enrichment Analysis (GSEA) revealed that the high-MDRG cluster had a relatively high degree of enrichment in the DNA replication pathway (Fig. 1G). Additionally, the Notch, Wnt, and TGF-β signaling pathways attracted our attention, as they are epithelial-to-mesenchymal transition-related pathways associated with enhanced migratory capabilities of tumor cells. The assessment of epithelial-to-mesenchymal transition scores for the two clusters also revealed that the high-MDRG cluster had a greater level of epithelial-to-mesenchymal transition (Fig. 1H). Further Weighted Gene Co-express Network Analysis (WGCNA), Gene Ontology (GO), and Kyoto Encyclopedia of Genes and Genomes (KEGG) enrichment analyses suggest that a high level of MDRGs may be strongly related to the enhanced proliferation and migration of LIHC cells (Fig. S5). We subsequently employed machine learning algorithms to construct an MDRG score for LIHC patients. For the convenience of clinical use, we further reduced the 10 MDRGs to 6 for constructing the signatures via the random forest algorithm (importance >0.01, Table S2). We employed a collective of 42 ensemble algorithms, and the predictive efficacy of these models was assessed using the training set, internal validation set, and external validation set. The results indicated that the random survival forest model had the best predictive ability (Fig. 1I). The risk scores, Kaplan–Meier curves, and the values for the area under the curve for the data set are displayed in Figure S6. We found that the risk scores derived from MDRGs for LIHC had good predictive performance, with patients with higher scores tending to have poorer prognosis. In addition, we conducted an online prediction platform integrated with the prognostic signature (mitusml.com:9545).

**Figure 1:**
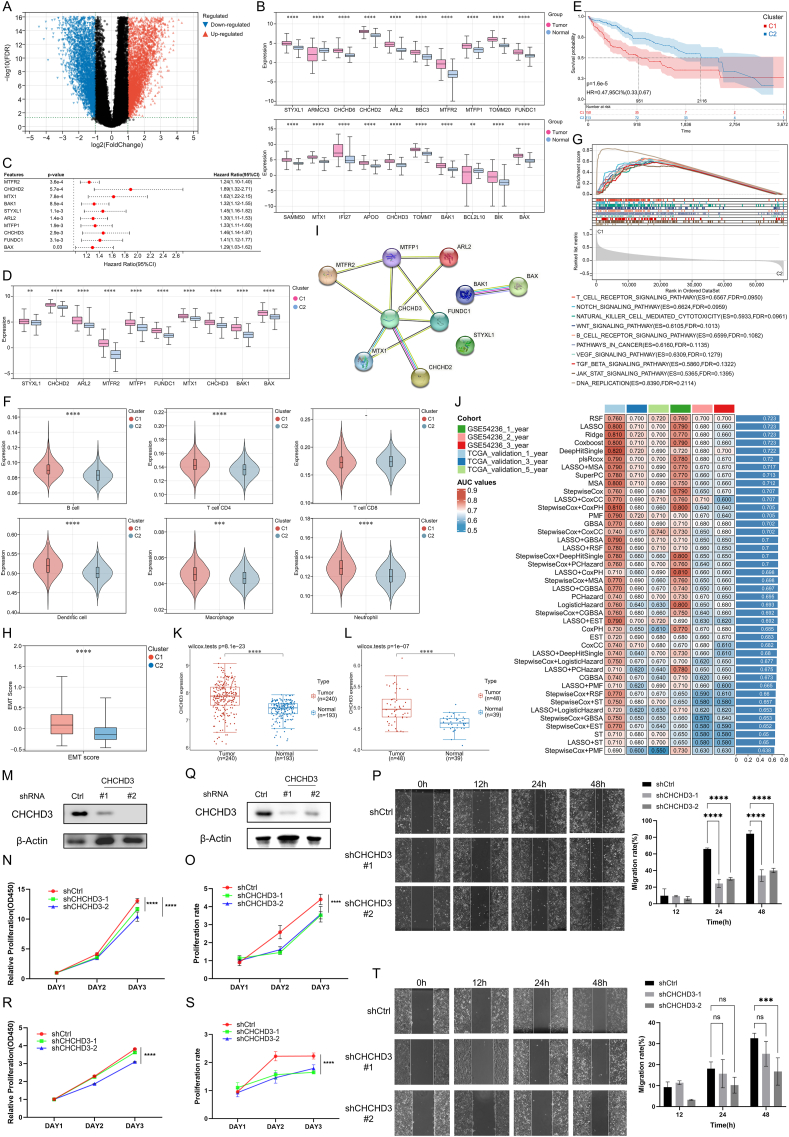
Exploration of mitochondrial dynamics-related genes (MDRGs) in liver hepatocellular carcinoma (LIHC). **(A)** Volcano plot for the selection of differentially expressed genes. **(B)** Expression levels of differentially expressed MDRGs in LIHC samples versus normal samples. **(C)** Forest plot of the 10 MDRGs with prognostic value. **(D)** Expression levels of the MDRGs used for clustering between the two subtypes. **(E)** The Kaplan–Meier curve shows the difference in patient prognosis between the two subtypes. **(F)** Levels of immune cells in the two subtypes. **(G)** The analysis of Gene Set Enrichment Analysis (GSEA). **(H)** Epithelial-to-mesenchymal transition (EMT) scores of the two subtypes. **(I)** The predictive performance of the 42 prognostic models constructed by MDRGs. **(J)** Protein–protein interaction network of 10 MDRGs. **(K, L)** Validation of *CHCHD3* expression levels in the GSE36376 (K) and GSE45267 (L) datasets. **(M)** Western blots for *CHCHD3* in Huh7 cells infected with control (Ctrl) or *CHCHD3* shRNAs (#1, #2). **(N)** CCK-8 proliferation assays in Huh7 cells transduced with shCtrl or shCHCHD3. **(O)** Cell proliferation was measured by counting Huh7 cells transduced with shCtrl or shCHCHD3. **(P)** Cell scratch assay with shCtrl and shCHCHD3 Huh7 cells for 0 h, 12 h, 24 h, and 48 h (left, representative images; right, quantification of the migrating area). Scale bar, 200 μm. **(Q)** Western blots for *CHCHD3* in HepG2 cells infected with control (Ctrl) or *CHCHD3* shRNAs (#1, #2). **(R)** CCK-8 proliferation assays in HepG2 cells transduced with shCtrl or shCHCHD3. **(S)** Cell proliferation was measured by counting HepG2 cells transduced with shCtrl or shCHCHD3. **(T)** Cell scratch assay with shCtrl and shCHCHD3 HepG2 cells for 0 h, 12 h, 24 h, and 48 h (left, representative images; right, quantification of the migrating area). Scale bar, 200 μm. C1 represents the high-MDRG cluster, and C2 represents the low-MDRG cluster. ∗*P* < 0.05, ∗∗*P* < 0.01, ∗∗∗*P* < 0.001, and ∗∗∗∗*P* < 0.0001.

*CHCHD3* was indicated as a key MDRG in LIHC (Fig. 1J; Table S3). The elevated expression of *CHCHD3*, as noted in the TCGA dataset, was also detected in GSE36376 (Fig. 1K) and GSE45267 (Fig. 1L). Immunohistochemistry also revealed *CHCHD3* up-regulation in LIHC (Fig. S7A). Single cell analysis also found that the *CHCHD3* was significantly up-regulated in tumor cells (Fig. S7B). We annotated the cells in the tumor tissues and identified a total of 10 clusters of cells, including hepatocytes (Fig. S7C). The hepatocytes were subdivided and annotated into three categories: normal cells (diploid), tumor cells (aneuploid), and unknown (not defined) (Fig. S7D). The expression of *CHCHD3* was greater in cancerous hepatocytes than in normal hepatocytes (Fig. S7E). This is consistent with the expression profile of *CHCHD3* in bulk RNA sequencing. In addition, molecular docking involving CHCHD3 and the drugs sorafenib, lenvatinib, regorafenib, and cabozantinib indicated the favorable prospects of CHCHD3 as a therapeutic target for LIHC (Fig. S7F–S7I; Table S4).

In the above section, we found the association between MDRG and tumor proliferation and migration. Bioinformatics analyses further suggested the significant positive correlation between *CHCHD3* and DNA replication and cell cycle pathway levels in LIHC, which indicates its close relationship with tumor cell proliferation (Fig. S8A and S8B). *CHCHD3* also showed a significant positive correlation with the levels of Wnt and TGF-β signaling pathways associated with epithelial-to-mesenchymal transition, suggesting its importance in tumor cell migration (Fig. S8C and S8D). Next, we proceeded with *in vitro* experiments to validate our findings. In Huh7 (Fig. 1M) and HepG2 (Fig. 1Q) cells, the expression of *CHCHD3* was knocked down, and western blotting results confirmed successful knockdown. In the CCK-8 assay, we observed a marked reduction in the proliferation of Huh7 (Fig. 1N and O) and HepG2 (Fig. 1R and S) cells following the knockdown of *CHCHD3*. The scratch assay revealed that in Huh7 cells with *CHCHD3* knockdown, the scratch healing level at 24 h and 48 h was reduced, and the cell migration rate was significantly decreased (Fig. 1P). In HepG2 cells with *CHCHD3* knockdown, the cell migration rate was decreased at 48 h (Fig. 1T). The experimental findings further reveal that the up-regulation of *CHCHD3* in LIHC might be intimately linked to the decline in proliferation and migration capabilities of tumor cells.

In summary, we identified two distinct mitochondrial dynamics-related clusters in LIHC, and the cluster with high MDRG expression had a poorer prognosis. The prognostic model developed based on MDRGs showed satisfactory performance. *CHCHD3* might be a potential biomarker for the diagnosis, management, and treatment of LIHC.

## CRediT authorship contribution statement

**Yuchen Zhang:** Visualization, Investigation, Writing – original draft, Methodology, Conceptualization, Software, Data curation, Validation, Formal analysis. **Jing Yuan:** Validation, Formal analysis, Visualization, Investigation, Writing – original draft, Methodology, Conceptualization, Software, Data curation. **Ning Zhang:** Software, Data curation, Validation, Formal analysis, Visualization, Investigation, Writing – original draft, Methodology, Conceptualization. **Yang Yang:** Writing – original draft, Methodology, Conceptualization, Software, Data curation, Validation, Formal analysis, Visualization, Investigation. **Chang Liu:** Validation, Data curation, Formal analysis, Investigation. **Xingyu Jiang:** Validation, Data curation, Formal analysis, Investigation. **Yue Wu:** Validation, Data curation, Formal analysis, Investigation. **Xingxing Ma:** Validation, Data curation, Formal analysis, Investigation. **Yinyin Xie:** Resources, Supervision, Funding acquisition, Validation, Investigation, Writing – review & editing, Project administration.

## Data availability

The MDRGs were collected from the mitochondrial dynamics pathway of the MitoCarta3.0 website (http://www.broadinstitute.org/mitocarta). The bulk RNA sequencing data and survival data were sourced from the TCGA and GTEx cohorts in UCSC Xena (https://xenabrowser.net/). The validation data and single-cell RNA sequencing data were downloaded from the GEO (https://www.ncbi.nlm.nih.gov/geo/) database. The protein–protein interaction network was retrieved from the STRING (https://cn.string-db.org/) database. The immunohistochemistry results were obtained from the Human Protein Atlas (HPA, https://www.proteinatlas.org/) database. The original version of all the figures was provided via the website Figshare (https://doi.org/10.6084/m9.figshare.28830620.v1).

## Funding

The work was funded by the University Research Fund of Anhui Medical University (China) (No. 2023xkj015), Natural Science Research Project of Anhui Educational Committee of China (No. 2024AH050756), and “Early Contact with Research” Training Program for the Clinical Medicine ("5+3" Integrated) Students (China) (No. 2024-ZQKY-104).

## Conflict of interests

An earlier version of this article was presented at IDDF 2024 Conference, Hong Kong, and the abstract was published in *Gut* (Zhang Y, Yuan J, Yang Y, et al. IDDF2024-ABS-0182 Exploring the role of mitochondrial dynamics-related genes in the molecular classification and prognostic value of liver hepatocellular carcinoma. *Gut*. 2024;73:A32. DOI:10.1136/gutjnl-2024-IDDF.23). No other conflicts of interest or competing interests are reported.
