# Supplementary material for: Comprehensive analysis and experimental verification of the role of mitochondrial dynamics-related genes in liver hepatocellular carcinoma
Source: Genes Dis. 2025 Sep 26;13(3):101872. doi: 10.1016/j.gendis.2025.101872 (PMC12818978; doi:10.1016/j.gendis.2025.101872)
Supplement: Multimedia component 1 [file mmc1.pdf]

Contents

Materials and methods ········ 2

Supplementary Figure 1. KM survival curves of the 10 differentially expressed MDRGs with significant prognostic relevance. ········ 7

Supplementary Figure 2. Identification of two mitochondrial dynamics-related LIHC subtypes. (A) Clustering heatmap of the two subtypes. CDF curves (B) and the area under the CDF curve (C) assist in the decision for the number of clusters. Intra-group consistency (D) and PCA (E) illustrate the rationality of the clustering. (C1, high-MDRG cluster; C2, low-MDRG cluster) ········ 8

Supplementary Figure 3. Analysis of immune infiltration between the two subtypes. (A) Abundances of six types of immune cells in patients. (B) Correlations between the six types of immune cells. (C) Differences in immune checkpoint-related genes between the two subtypes. (C1, high-MDRG cluster; C2, low-MDRG cluster; *$p < 0.05$, **$p < 0.01$, ***$p < 0.001$, and ****$p < 0.0001$) ········ 9

Supplementary Figure 4. Mutation landscapes of the two LIHC subtypes. (A) The landscape of base mutations in LIHC patients. (B) Waterfall plot showing the gene mutations of the two subtypes. Details of the mutation landscape for the high-MDRG cluster (C) and low-MDRG cluster (E). Correlations between mutated genes in the high-MDRG cluster (D) and low-MDRG cluster (F). (C1, high-MDRG cluster; C2, low-MDRG cluster) ········ 10

Supplementary Figure 5. WGCNA analysis. (A) Selecting the most appropriate soft threshold. (B) Gene dendrogram of WGCNA. (C) Correlations between modules and LIHC subtypes. (D) Diagram depicting the correlation between the green module and the high-MDRG cluster via scatter plot. GO analysis (E) and KEGG analysis (F) of subtype hub genes. (C1, high-MDRG cluster; C2, low-MDRG cluster) ········ 11

Supplementary Figure 6. Heatmap of the risk score and modeling of gene correlations (A), KM curve (B), and ROC curves (C) in the TCGA cohort. Heatmap of the risk score and modeling gene correlation (D), KM curve (E), and ROC curves (F) in the GEO cohort. ········ 12

Supplementary Figure 7. Determination of the key gene *CHCHD3*. (A) Immunohistochemistry of *CHCHD3* in LIHC and control samples. (B) Differential expression of *CHCHD3* in tumor tissue versus normal tissue from GSE242889. (C) Cell annotation in tumor tissue. (D) Identification of tumor cells and normal hepatocytes. (E) Expression levels of *CHCHD3* in tumor cells and normal hepatocytes. Molecular docking analysis of CHCHD3 with sorafenib (F), lenvatinib (G), regorafenib (H), and cabozantinib (I). · 13

Supplementary Figure 8. The expression of *CHCHD3* correlates with the DNA replication pathway (A), cell cycle pathway (B), Wnt signaling pathway (C), and TGF-β signaling pathway (D) in LIHC. (***$p < 0.001$) ········ 14

Supplementary Table 1. The clinical features of patients of two clusters. ········ 15

Supplementary Table 2. Importance for the 10 MDRGs calculated by RF algorithm. ········ 16

Supplementary Table 3. Scores for key MDRGs using the MCC and Degree algorithms. ········ 17

Supplementary Table 4. The binding energy of CHCHD3 with four drugs of LIHC. ········ 18

Supplementary Table 5. The primer sequences of *CHCHD3*. ········ 19

Materials and methods

Data collection

In this study, MDRGs were collected from the mitochondrial dynamics pathway of the MitoCarta3.0 website [1]; bulk RNA-seq data and survival data of LIHC patients were sourced from the TCGA (The Cancer Genome Atlas) database and the GSE36376 and GSE45267 datasets from the GEO (Gene Expression Omnibus) database. We also supplemented control samples from the GTEx (Genotype-Tissue Expression) database. The immunohistochemistry results were downloaded from the HPA (The Human Protein Atlas) database.

Screening for differentially expressed MDRGs and assessment of their prognostic value

The "limma" package within the R program was utilized to perform differential expression analysis. The criteria for significance were established as a false discovery rate (FDR) below 0.05 and a log2(fold change) magnitude exceeding 1. The prognostic value was further assessed via the "survival" R package and univariate Cox regression.

Identification of LIHC subtypes

Consensus cluster analysis of LIHC patients was performed via the "ConsensusClusterPlus" package in R software, with a maximum of 6 clusters, an 80% sampling ratio, and the sampling process was repeated 10 times. We utilized the "stats" package in R to perform principal component analysis (PCA), aiming to delve deeper into the classification outcomes.

Immune-related analysis and mutation landscape of LIHC subtypes

We utilized the TIMER algorithm [2] to evaluate the levels of six common immune cell types in patients, and the expression of immune checkpoint-related genes further demonstrated the differences in immune infiltration among subtypes. Mutation analysis was carried out using the "maftools" package within the R programming environment, revealing distinct mutation landscapes among different MDRG-related LIHC subtypes.

Enrichment analysis

Initial exploration of the differences in biological functions between subtypes was conducted through gene set enrichment analysis (GSEA) using the GSEA software. The epithelial to mesenchymal transition (EMT) score was computed via the R package "imogimap" [3]. Weighted correlation network analysis (WGCNA) was carried out via the R package "WGCNA" [4] to identify hub genes associated with subtypes. The "ClusterProfiler" R package was employed for conducting Gene Ontology (GO) and Kyoto Encyclopedia of Genes and Genomes (KEGG) enrichment analysis. The sensitivity for identifying co-expression modules was set to 3, the smallest module dimension was established at 30, and the merging threshold was set to 0.25 in

the WGCNA analysis. When identifying hub genes, the gene significance (GS) and module membership (MM) thresholds were set to 0.5 and 0.8, respectively.

Construction of a prognostic model based on MDRGs for LIHC

The TCGA cohort was allocated into training and internal validation subsets with a proportion of 70% and 30%, respectively. An independent dataset (GSE54236) was utilized as the external validation set. The "TDM" R package was employed to eliminate batch effects between the TCGA dataset and the GSE54236 dataset while preserving biological effects [5]. The MDRGs selected for modeling were identified via the Random Forest (RF) algorithm from the R package "randomForestSRC" [6]. Subsequently, 18 basic algorithms including Least Absolute Shrinkage and Selection Operator (LASSO) and Ridge from the "glmnet" package in R; the Coxboost algorithm from the "CoxBoost" package; the plsRcox algorithm from the "plsRcox" package; the SuperPC algorithm from the "superpc" package; the StepwiseCox algorithm from the "survival" package; the Random Survival Forest (RSF) algorithm, Gradient Boosting Survival Analysis (GBSA) algorithm, Component-wise Gradient Boosting Survival Analysis (CGBSA) algorithm, Extra Survival Trees (EST) algorithm, Minlip Survival Analysis (MSA) algorithm, and Survival Tree (ST) algorithm from the "scikit-survival" library in Python [7]; and the DeepHitSingle algorithm, CoxCC algorithm, CoxPH algorithm, PMF algorithm, PCHazard algorithm, and LogisticHazard algorithm from the "pycox" library, were combined into 42 ensemble algorithms for the establishment of prognostic models. The R package "pROC" was utilized to compute the time-dependent area under the curve (AUC) associated with receiver operating characteristic (ROC) analyses [8]. The online prediction platform equipped with the prognostic model was constructed using the Streamlit library in Python.

Molecular docking analysis

The structure of the CHCHD3 protein was predicted utilizing the AlphaFold website, and while the structural data for the pharmaceutical compounds were retrieved from the PubChem repository. Subsequent to the elimination of water molecules and the incorporation of polar hydrogen atoms into both the protein and the pharmaceutical molecules, the binding affinity and interaction patterns between the drugs and CHCHD3 were evaluated with AutoDock Vina software.

Single-cell analysis

The scRNA-seq data were retrieved from the GSE242889 entry within the GEO repository. The single-cell workflow was created via the R package "Seurat" [9]· and cell annotation was facilitated via the CellMarker [10] and Enrichr [11] websites. The "copycat" package was used to identify malignant cells [12].

Cell culture

HepG2 and Huh7 cells were cultured with DMEM medium (Biosharp, BL304A) containing 10% fetal bovine serum (BioChannel) and 1% penicillin-streptomycin solution (Biosharp, BL505A) in 5% CO^2 incubator at 37℃.

Construction of CHCHD3 shRNA expressing plasmids

The target sequences were from the MISSION shRNA Library (Sigma Aldrich) showed in **Supplementary Table 5**. The primers formed double-stranded DNA by annealing. PLKO plasmid were digested with EcoRI and AgeI, and recovered large fragments with gel extraction kit. PCR products ligated into above digested plasmid PLKO through T4 ligase (Takara, 2011A).

Construction of stably knockdown cell lines

HEK293T cells were inoculated into 6-well plates with a density of about 80% before transfection. shRNA expressing plasmid and package plasmids (psPAX2 and pMD2.G) were added to the bottom of EP tube according to the mass ratio of 3:2:1 (total amount of 4μg per well), and then 250μl of serum-reducing medium was added. A new EP tube was prepared by adding 250μl of serum-reducing medium and 8μL of transfection reagent. The mixture was thoroughly combined and incubated at room temperature for 20min before added to HEK293T cells, followed by gentle shaking. After 6-8h, the existing culture medium was removed and replaced with 2 mL of DMEM comprehensive culture medium. The timing was stared immediately after the medium exchange. The virus-containing supernatant was collected at 24h and 48h post-transfection. Notably, most of the HEK293T cell had died by the 48-hour collection point. The collected supernatant was centrifuged at 4000 rpm for 10min.

When the HepG2/Huh7 cells in 6-well plate reached 50% confluency, virus and polybrene (10μg/mL) were added. After 24 hours of infection, the medium was replaced with fresh DMEM complete medium for an additional 24h. At 48 hours post-infection, puromycin was added at a concentration of 3μg/ml. After 48 hours of selection, all non-infected control cells had died. The viable infected cells were harvested, and western blotting (WB) was conducted to evaluate the efficiency of gene knockdown for the target gene.

Western blotting

HepG2/Huh7 cells were washed twice with PBS and then placed on ice for further manipulation. 1 mL of pre-cooled 1× PBS was added, and the cells were thoroughly scraped from the dish using a cell scraper. The cell suspension was then transferred to a centrifuge tube and centrifuged at 3000 rpm for 5 minutes, after which the supernatant was discarded. RIPA lysis buffer was added, and the cells were lysed on ice for 30 minutes. The lysate was centrifuged at 12,000 rpm for 20 minutes at 4°C, and the supernatant was collected for BCA quantification. The appropriate amount of loading buffer was added to the sample, and it was heated at 95°C

for 5 minutes. A 12% SDS-PAGE gel was prepared, and after electrophoresis, the proteins were transferred to a membrane using a wet transfer method at a constant current of 300 mA for 2 hours. After the transfer, the membrane was blocked with 5% skim milk at room temperature for 1 hour. The primary antibodies, including CHCHD3 (Proteintech, 25625-1-AP, 1:4000) and β-Actin (Proteintech, 66009-1-1g, 1:10000), were added and incubated overnight at 4°C. The membrane was washed with 0.1% TBST for 5 minutes each time, repeated three times. The secondary antibody was then added and incubated at room temperature for 1 hour, followed by washing with 0.1% TBST three times, 5 minutes each. Finally, the signal was detected using ECL detection reagent.

CCK-8 assay

HepG2/Huh7 cells were seeded into 96-well plates at a density of 5,000 cells per well and incubated for 24 hours. After the incubation period, cell viability was assessed using the Cell Counting Kit-8 (CCK-8) (TOPSCIENCE, C0005). The CCK-8 reagent was added to each well, and the cells were incubated for 1 hour. The optical density (OD) at 450 nm was measured using a microplate reader, and relative cell viability was calculated based on the OD values.

Cell scratch assay

Straight lines were drawn every 0.5 cm on the back of a six-well plate to serve as reference markers. Cells were seeded at a density of 4 × 10^5 cells per well. Once the cells reached confluency, the medium was replaced with DMEM containing 1% fetal bovine serum. A wound was created in the cell monolayer by scratching the surface using a 200 μL pipette tip, perpendicular to the reference lines. The plate was then incubated in a 5% CO^2 incubator at 37°C. Images of the wound area were captured at 0-, 12-, 24-, and 48-hours post-scratch. Wound closure was analyzed using ImageJ software.

Statistical analysis

In bioinformatics analysis, statistical analysis was performed via R software. The Wilcoxon test was employed to evaluate differences between the two groups, whereas the Kruskal–Wallis test was utilized to assess differences across multiple groups. The Kaplan–Meier curves were tested via the log-rank test. The data from the CCK-8 proliferation assay and the cell scratch assay were analyzed via GraphPad Prism 10 software, with differences tested via two-way analysis of variance (ANOVA). A p value below the 0.05 threshold was indicative of statistical significance.

References

1. Rath S, Sharma R, Gupta R, Ast T, Chan C, Durham TJ, et al. MitoCarta3.0: an updated mitochondrial proteome now with sub-organelle localization and pathway annotations. Nucleic Acids Res. 2021; 49: D1541-D7.
2. Li B, Severson E, Pignon J-C, Zhao H, Li T, Novak J, et al. Comprehensive analyses of tumor immunity:

implications for cancer immunotherapy. Genome Biol. 2016; 17: 174.
3. Bozorgui B, Kong EK, Luna A, Korkut A. Mapping the functional interactions at the tumor-immune checkpoint interface. Commun Biol. 2023; 6: 462.
4. Langfelder P, Horvath S. WGCNA: an R package for weighted correlation network analysis. BMC Bioinformatics. 2008; 9: 559.
5. Thompson JA, Greene CS. Training Distribution Matching (TDM) R Package: Zenodo.
6. Hemant I, Udaya BK, Eugene HB, Michael SL. Random survival forests. The Annals of Applied Statistics. 2008; 2: 841-60.
7. Pölsterl S. scikit-survival: A Library for Time-to-Event Analysis Built on Top of scikit-learn. Journal of Machine Learning Research. 21: 1-6.
8. Robin X, Turck N, Hainard A, Tiberti N, Lisacek F, Sanchez J-C, et al. pROC: an open-source package for R and S+ to analyze and compare ROC curves. BMC Bioinformatics. 2011; 12: 77.
9. Hao Y, Stuart T, Kowalski MH, Choudhary S, Hoffman P, Hartman A, et al. Dictionary learning for integrative, multimodal and scalable single-cell analysis. Nat Biotechnol. 2024; 42: 293-304.
10. Zhang X, Lan Y, Xu J, Quan F, Zhao E, Deng C, et al. CellMarker: a manually curated resource of cell markers in human and mouse. Nucleic Acids Res. 2019; 47: D721-D8.
11. Kuleshov MV, Jones MR, Rouillard AD, Fernandez NF, Duan Q, Wang Z, et al. Enrichr: a comprehensive gene set enrichment analysis web server 2016 update. Nucleic Acids Res. 2016; 44: W90-W7.
12. Gao R, Bai S, Henderson YC, Lin Y, Schalck A, Yan Y, et al. Delineating copy number and clonal substructure in human tumors from single-cell transcriptomes. Nat Biotechnol. 2021; 39: 599-608.

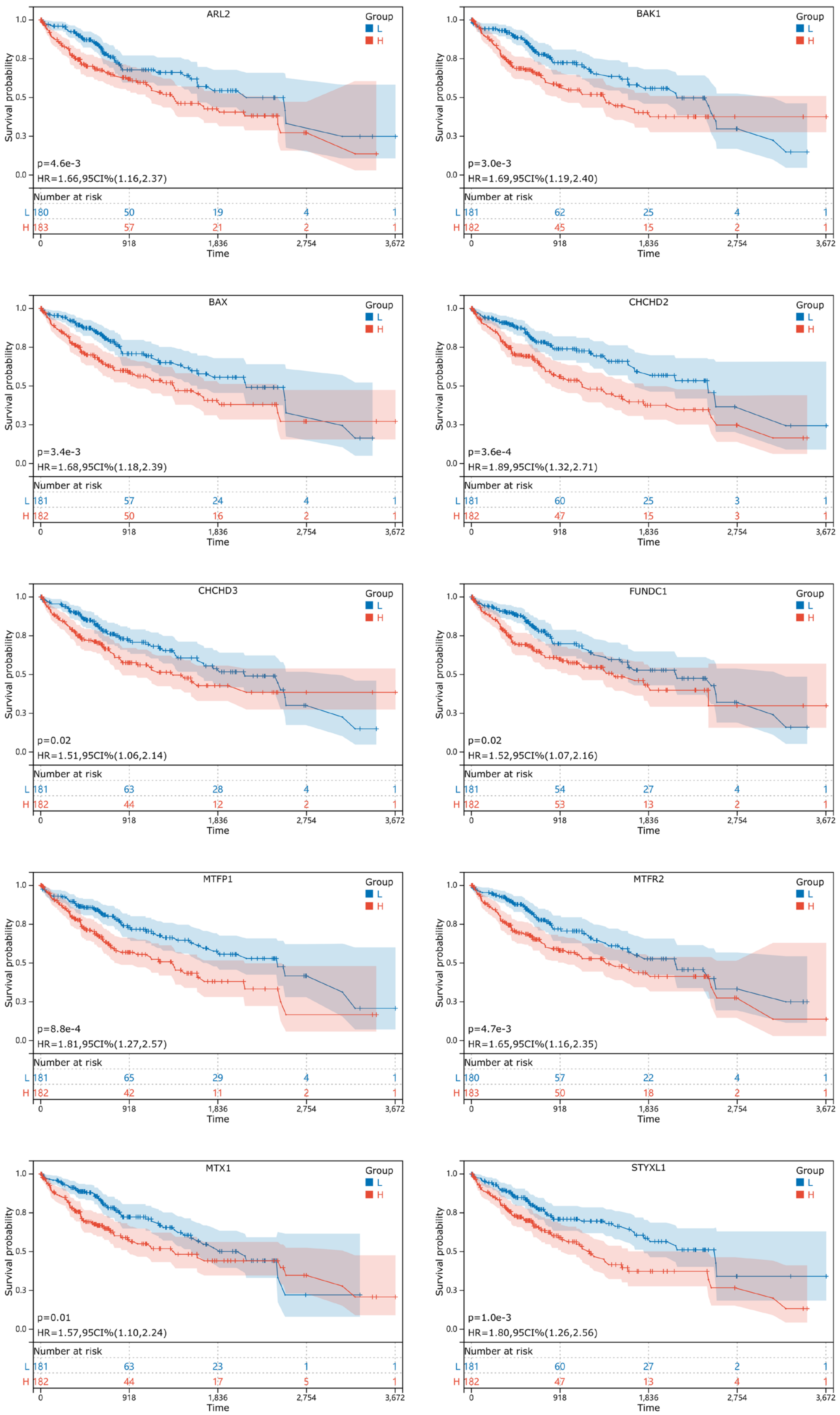

Supplementary Figure 1. KM survival curves of the 10 differentially expressed MDRGs with significant prognostic relevance.

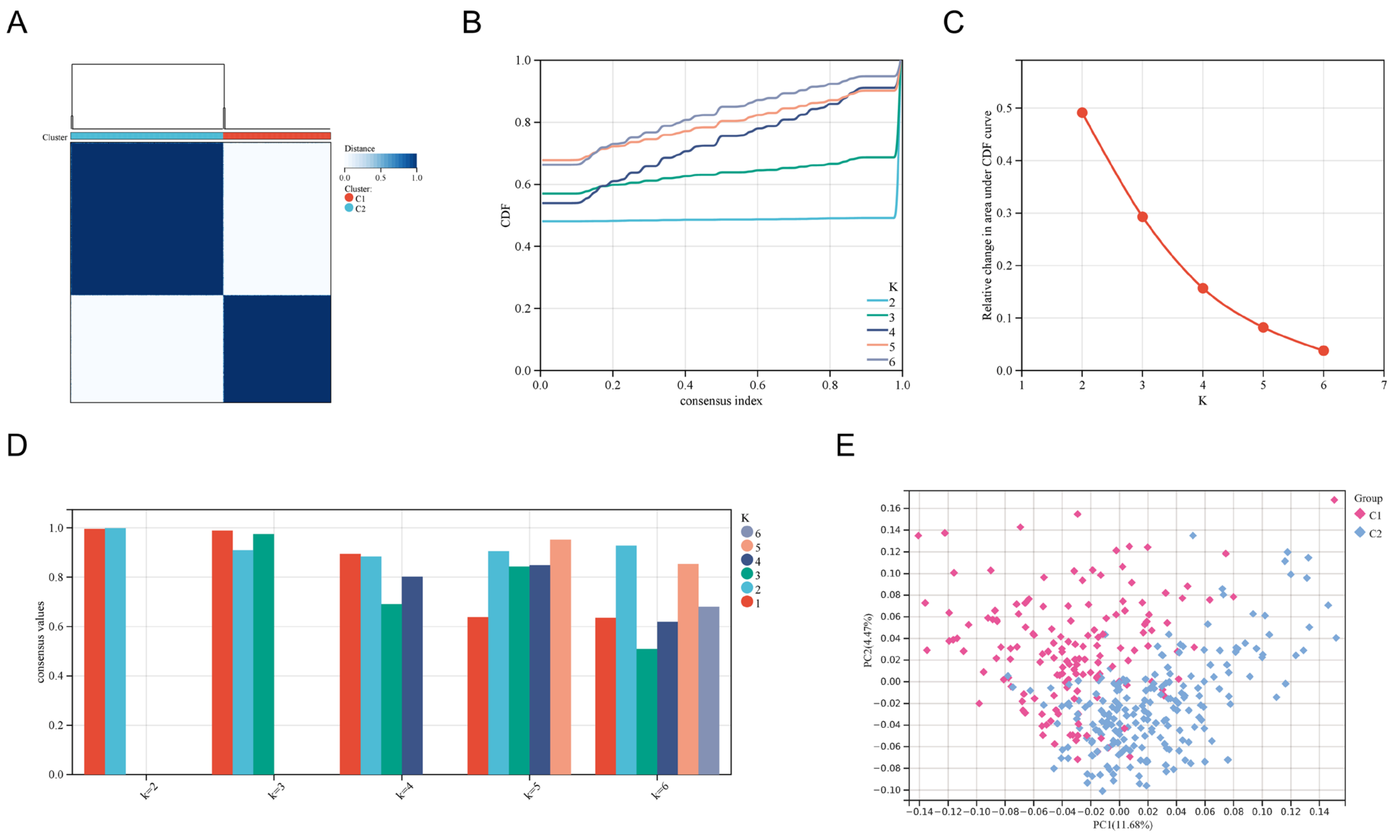

Supplementary Figure 2. Identification of two mitochondrial dynamics-related LIHC subtypes. (A) Clustering heatmap of the two subtypes. CDF curves (B) and the area under the CDF curve (C) assist in the decision for the number of clusters. Intra-group consistency (D) and PCA (E) illustrate the rationality of the clustering. (C1, high-MDRG cluster; C2, low-MDRG cluster)

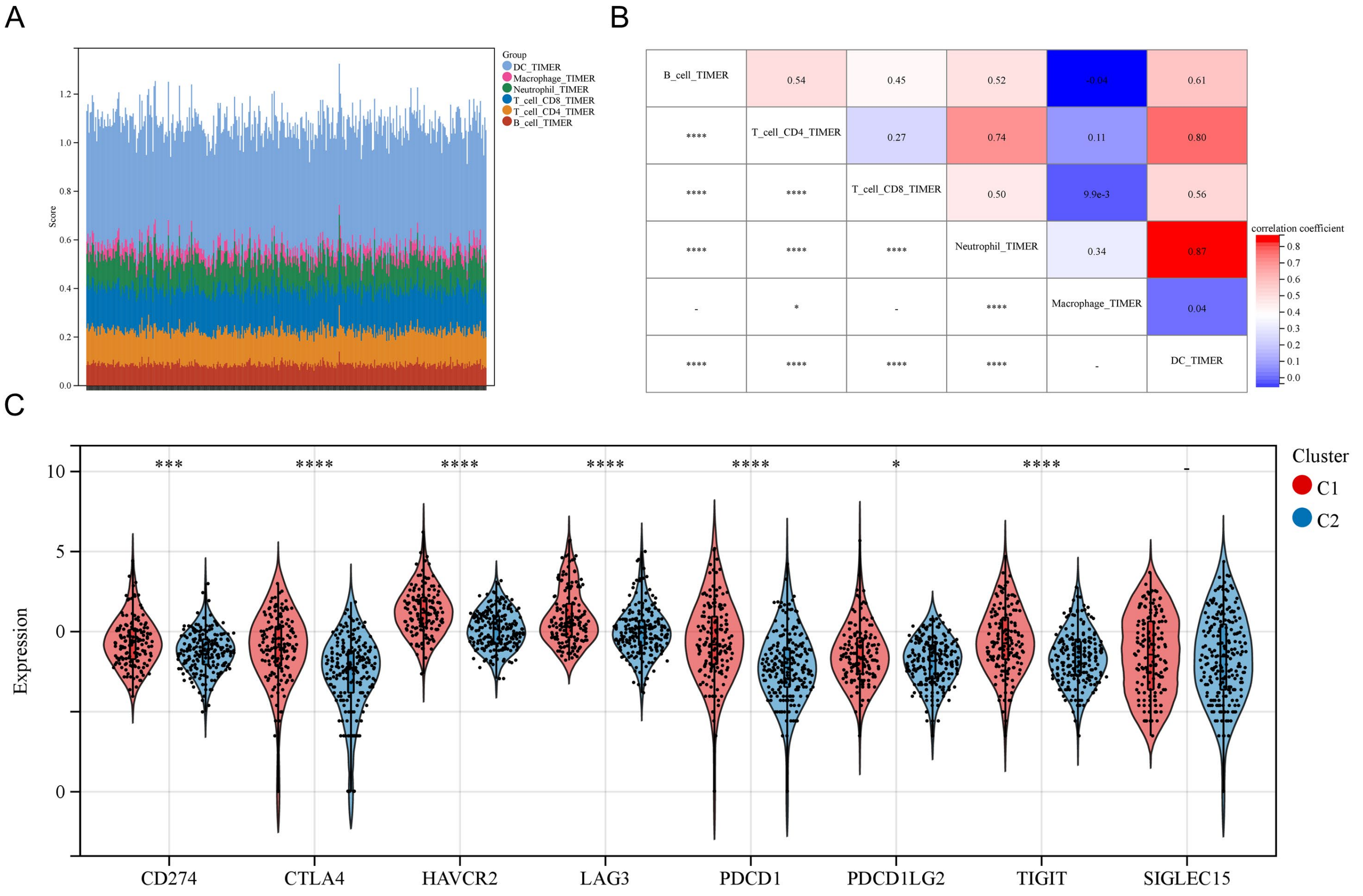

Supplementary Figure 3. Analysis of immune infiltration between the two subtypes. (A) Abundances of six types of immune cells in patients. (B) Correlations between the six types of immune cells. (C) Differences in immune checkpoint-related genes between the two subtypes. (C1, high-MDRG cluster; C2, low-MDRG cluster; *$p < 0.05$, **$p < 0.01$, ***$p < 0.001$, and ****$p < 0.0001$)

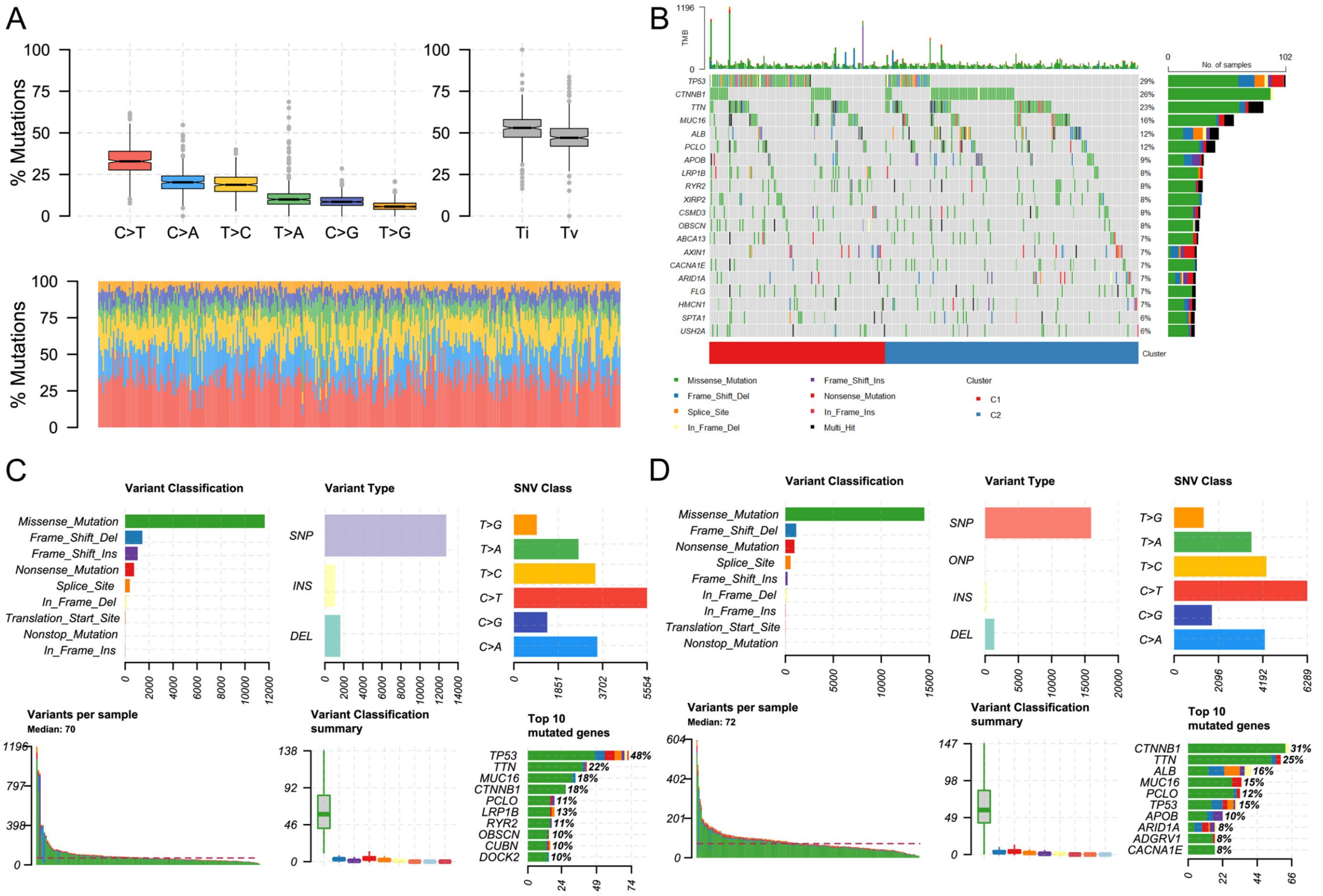

Supplementary Figure 4. Mutation landscapes of the two LIHC subtypes. (A) The landscape of base mutations in LIHC patients. (B) Waterfall plot showing the gene mutations of the two subtypes. Details of the mutation landscape for the high-MDRG cluster (C) and low-MDRG cluster (E). Correlations between mutated genes in the high-MDRG cluster (D) and low-MDRG cluster (F). (C1, high-MDRG cluster; C2, low-MDRG cluster)

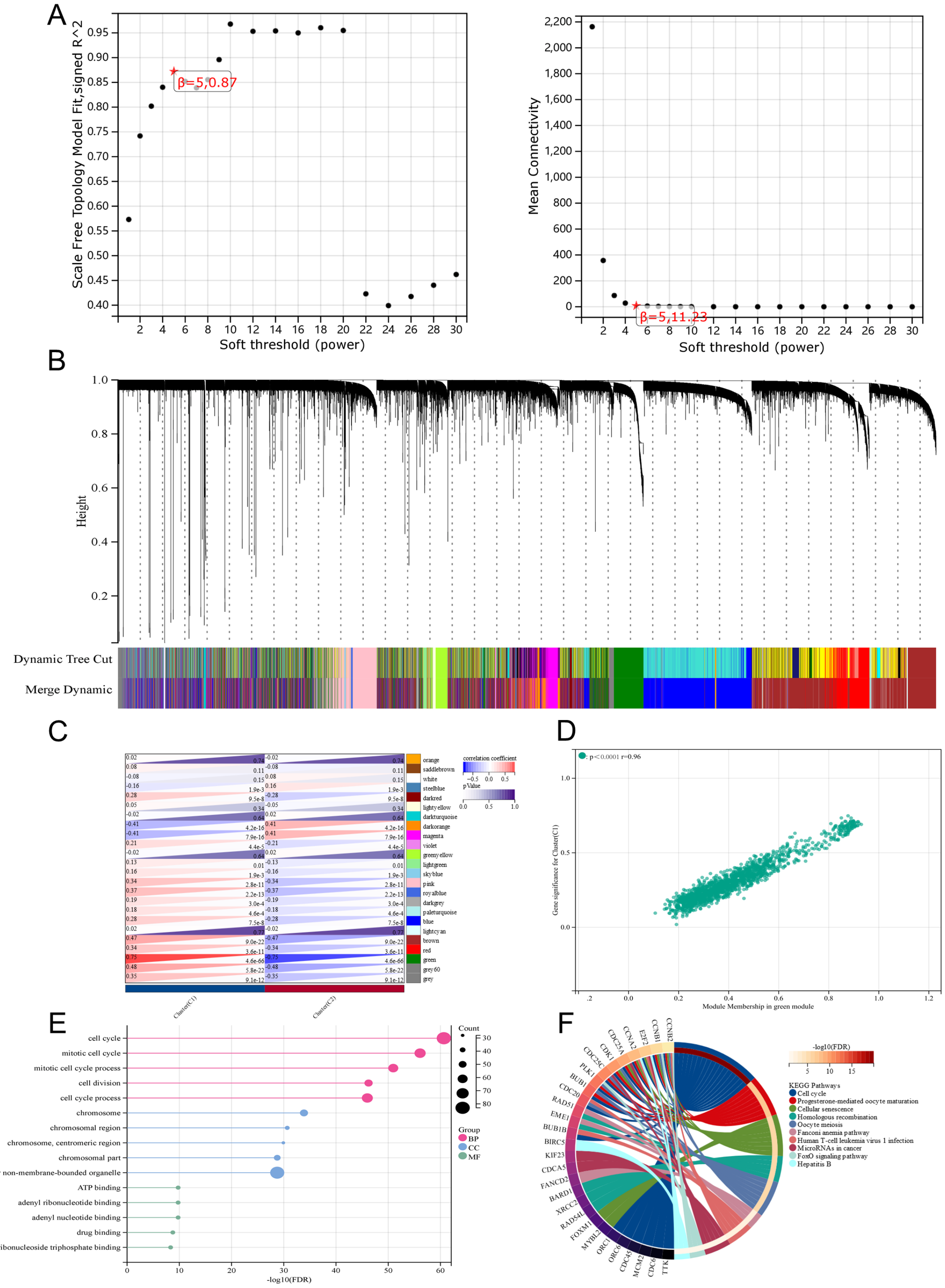

Supplementary Figure 5. WGCNA analysis. (A) Selecting the most appropriate soft threshold. (B) Gene dendrogram of WGCNA. (C) Correlations between modules and LIHC subtypes. (D) Diagram depicting the correlation between the green module and the high-MDRG cluster via scatter plot. GO analysis (E) and KEGG analysis (F) of subtype hub genes. (C1, high-MDRG cluster; C2, low-MDRG cluster)

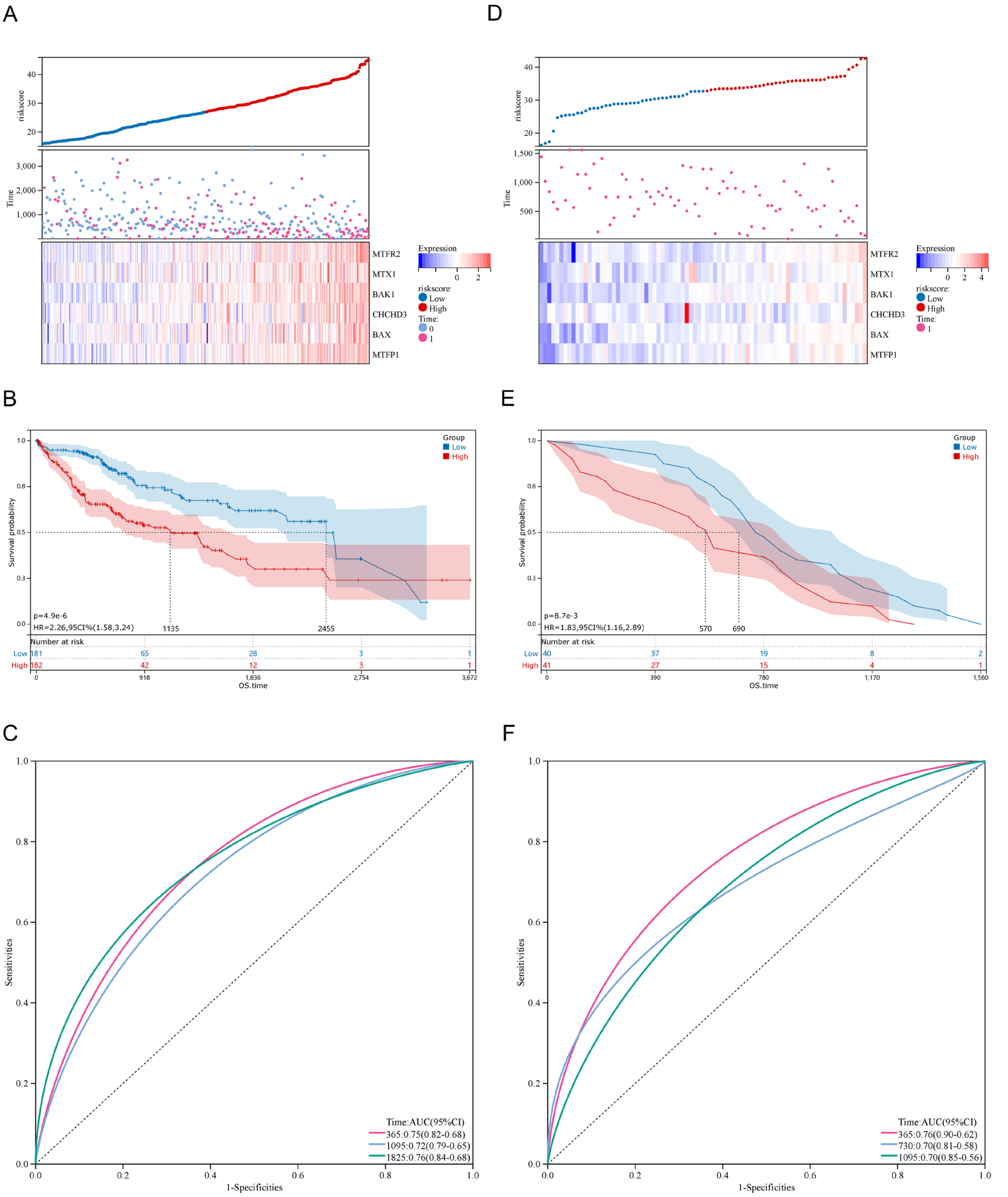

Supplementary Figure 6. Heatmap of the risk score and modeling of gene correlations (A), KM curve (B), and ROC curves (C) in the TCGA cohort. Heatmap of the risk score and modeling gene correlation (D), KM curve (E), and ROC curves (F) in the GEO cohort.

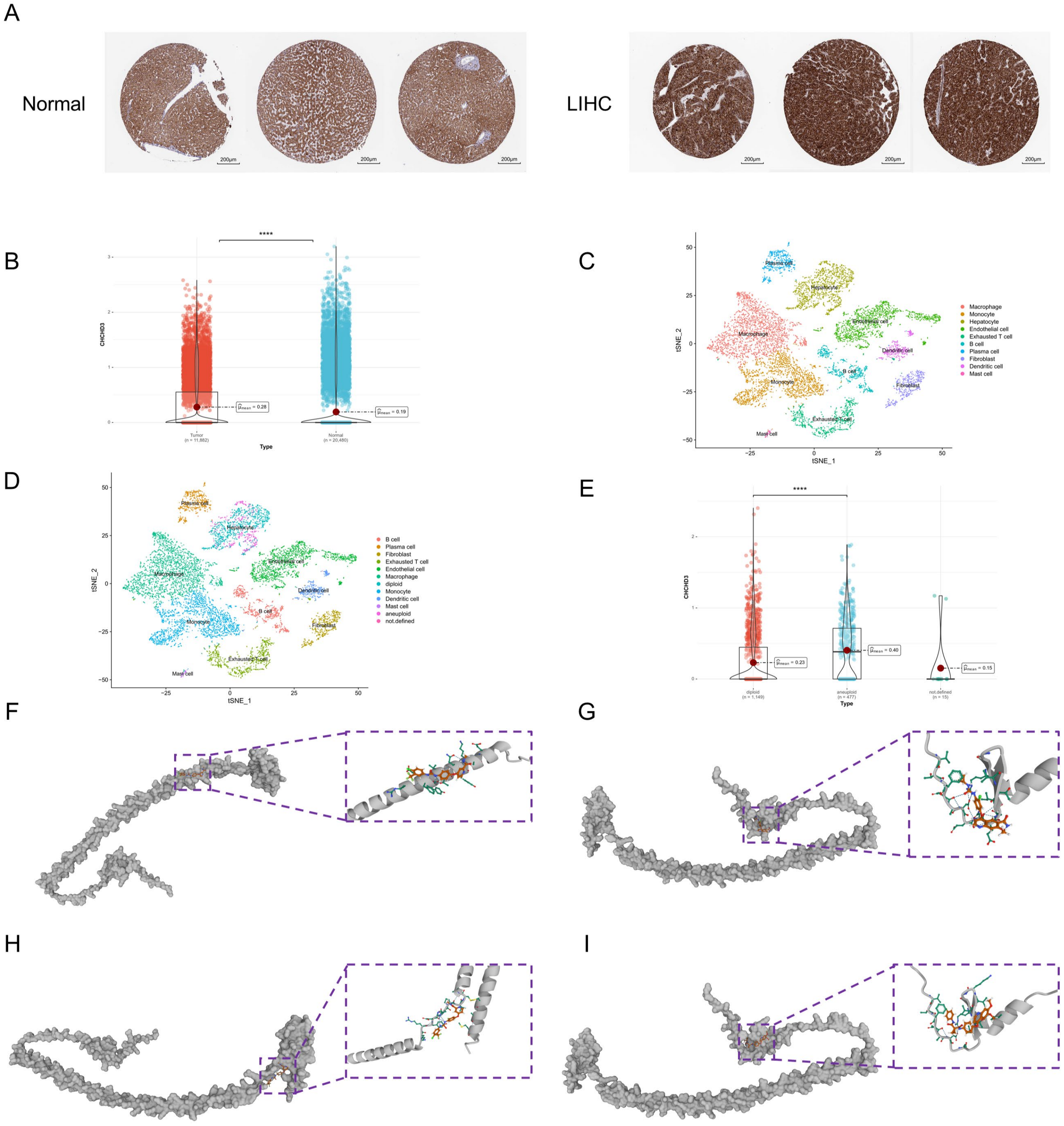

Supplementary Figure 7. Determination of the key gene *CHCHD3*. (A) Immunohistochemistry of *CHCHD3* in LIHC and control samples. (B) Differential expression of *CHCHD3* in tumor tissue versus normal tissue from GSE242889. (C) Cell annotation in tumor tissue. (D) Identification of tumor cells and normal hepatocytes. (E) Expression levels of *CHCHD3* in tumor cells and normal hepatocytes. Molecular docking analysis of CHCHD3 with sorafenib (F), lenvatinib (G), regorafenib (H), and cabozantinib (I).

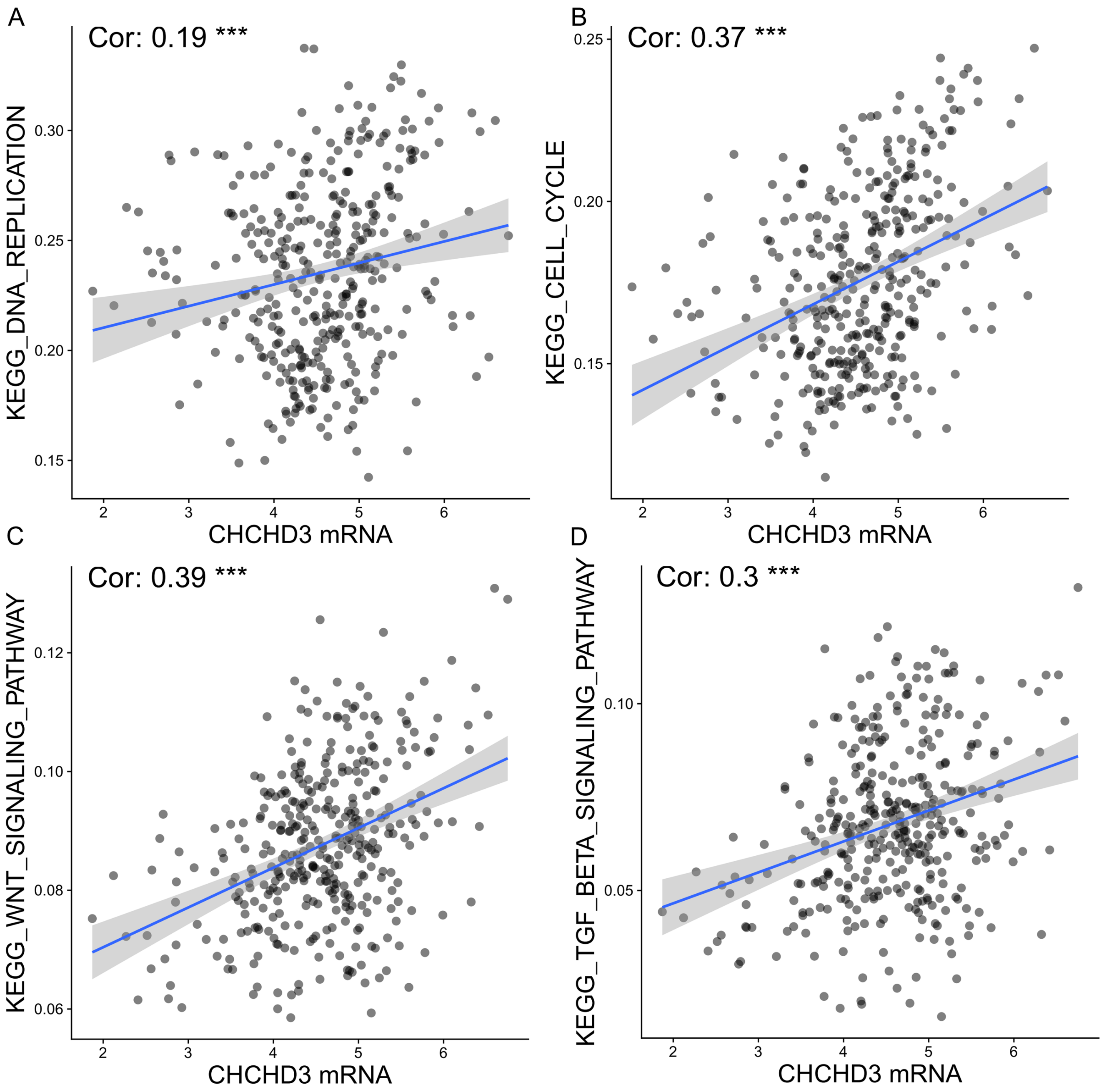

Supplementary Figure 8. The expression of *CHCHD3* correlates with the DNA replication pathway (A), cell cycle pathway (B), Wnt signaling pathway (C), and TGF-β signaling pathway (D) in LIHC. (***$p < 0.001$)

Supplementary Table 1. The clinical features of patients of two clusters.

Characteristics	C1(N=150)	C2(N=213)	Total(N=363)	*p* value
**Age**				0.08
Mean±SD	58.37±13.15	60.48±13.39	59.61±13.31	
Median[min-max]	60.00[18.00,83.00]	62.00[16.00,90.00]	61.00[16.00,90.00]	
**Gender**				0.19
Female	55(15.15%)	63(17.36%)	118(32.51%)	
Male	95(26.17%)	150(41.32%)	245(67.49%)	
**Race**				0.17
American Indian or Alaska Native	0(0%)	1(0.28%)	1(0.28%)	
Asian	70(19.28%)	84(23.14%)	154(42.42%)	
Black or African American	8(2.20%)	9(2.48%)	17(4.68%)	
White	71(19.56%)	110(30.30%)	181(49.86%)	
NA	1(0.28%)	9(2.48%)	10(2.75%)	
**Stage**				0.03
Stage I	53(14.60%)	115(31.68%)	168(46.28%)	
Stage II	40(11.02%)	44(12.12%)	84(23.14%)	
Stage III	1(0.28%)	2(0.55%)	3(0.83%)	
Stage IIIA	35(9.64%)	28(7.71%)	63(17.36%)	
Stage IIIB	5(1.38%)	3(0.83%)	8(2.20%)	
Stage IIIC	6(1.65%)	3(0.83%)	9(2.48%)	
Stage IV	0(0%)	1(0.28%)	1(0.28%)	
Stage IVA	0(0%)	1(0.28%)	1(0.28%)	
Stage IVB	1(0.28%)	1(0.28%)	2(0.55%)	
NA	9(2.48%)	15(4.13%)	24(6.61%)	
**Grade**				＜0. 0001
G1	12(3.31%)	43(11.85%)	55(15.15%)	
G2	62(17.08%)	113(31.13%)	175(48.21%)	
G3	67(18.46%)	50(13.77%)	117(32.23%)	
G4	7(1.93%)	4(1.10%)	11(3.03%)	
NA	2(0.55%)	3(0.83%)	5(1.38%)	

Supplementary Table 2. Importance for the 10 MDRGs calculated by RF algorithm.

Gene	Importance
MTFR2	0.0585
MTX1	0.0205
BAK1	0.018
CHCHD3	0.0178
BAX	0.0148
MTFP1	0.014
CHCHD2	0.0098
FUNDC1	0.0094
STYXL1	0.0094
ARL2	0.0067

Supplementary Table 3. Scores for key MDRGs using the MCC and Degree algorithms.

Gene	MCC	Degree
CHCHD3	9	12
MTFP1	6	8
FUNDC1	4	6
MTFR2	2	4
MTX1	2	4
ARL2	2	4
CHCHD2	1	2
BAX	1	2
BAK1	1	2

Supplementary Table 4. The binding energy of CHCHD3 with four drugs of LIHC.

**Drug**	**Binding energy (kcal/mol)**
Sorafenib	-6.314
Lenvatinib	-5.293
Regorafenib	-6.177
Cabozantinib	-6.280

Supplementary Table 5. The primer sequences of *CHCHD3*.

CHCHD3	Sequence
**shCHCHD3-1**	
Forward	5′ -CCGGCCTCAGTTTCTGATGAAGAAT CTCGAGATTCTTCATCAGAAACTGAGGTTTTTG-3′
Reverse	5′ -AATTCAAAAACCTCAGTTTCTGATGAAGAATCTCGAGATTCTTCATCAGAAACTGAGGCCGG-3′
**shCHCHD3-2**	
Forward	5′ -CCGGGCGGTATTCTGGTGCTTATGGCTCGAGCCATAAGCACCAGAATACCGCTTTTTG-3′
Reverse	5′ -AATTCAAAAAGCGGTATTCTGGTGCTTATGGCTCGAGCCATAAGCACCAGAATACCGCCCGG-3′
